# Health-Seeking Behavior for Erectile Dysfunction in Methadone Maintenance Treatment Patients

**DOI:** 10.3390/ijerph16214249

**Published:** 2019-11-01

**Authors:** Fitri Fareez Ramli, Ahmad Nazrun Shuid, Rashidi Mohamed Pakri Mohamed, Tg Mohd Ikhwan Tg Abu Bakar Sidik, Isa Naina Mohamed

**Affiliations:** 1 Department of Pharmacology, Faculty of Medicine, Universiti Kebangsaan Malaysia, Cheras 56000, Kuala Lumpur, Malaysia; fitrifareez@ppukm.ukm.edu.my (F.F.R.); anazrun@yahoo.com (A.N.S.); 2 Department of Family Medicine, Faculty of Medicine, Universiti Kebangsaan Malaysia, Cheras 56000, Kuala Lumpur, Malaysia; drrashidi5377@yahoo.com.my; 3 Pharmacoepidemiology and Drug Safety Unit, Department of Pharmacology, Faculty of Medicine, Universiti Kebangsaan Malaysia, Cheras 56000, Kuala Lumpur, Malaysia; tgikhwancrc@gmail.com

**Keywords:** health-seeking behavior, methadone, erectile dysfunction

## Abstract

*Background*: Erectile dysfunction (ED) is commonly associated with methadone usage. However, little data is known regarding the health-seeking behavior for ED in the methadone maintenance treatment (MMT) population. This study aimed to determine the health-seeking behavior of MMT patients with ED who perceived themselves as having ED. We aimed to assess the attitudes and health-seeking behavior, the effectiveness of the treatment and the factors associated with treatment-seeking behavior. *Methods*: This was an observational questionnaire-based study. Patients were first screened for ED (*n* = 154) using the International Index of Erectile Function-5 (IIEF-5). Fifty patients with ED were evaluated for health-seeking behavior for ED. *Results*: More than half of the patients who thought they had ED (78%) believed their sex life was affected. Most patients (48%) did not seek any information regarding ED. Education level (*p* = 0.017) and marital status (*p* = 0.008) were predictive factors of health-seeking behavior. *Conclusions*: The health-seeking rate among MMT patients with ED needs to be improved. Measures to increase awareness of ED in MMT patients should be taken to overcome the barrier to health-seeking behavior. Health practitioners should take action to screen ED in this population to increase the detection rate and offer appropriate management according to the patients’ needs.

## 1. Introduction

Methadone is a prescribed opioid that is indicated for the treatment of opioid use disorder. It has been widely used as a replacement therapy for opioid addiction in a program known as methadone maintenance treatment (MMT). This program has been proven to reduce illicit opioids use, crime rate, human deficiency virus (HIV) risk behavior as well as improve the social function and the health of MMT patients [1,2]. Despite its proven effectiveness, methadone is associated with several adverse effects, such as erectile dysfunction (ED).

ED is defined as the inability to accomplish or sustain penile erection sufficient for satisfactory sexual intercourse [3]. The risk factors for ED are multifactorial. Long-term use of opioids, including methadone, is one of the well-known risk factors associated with ED [4,5]. In opioid users, the mechanism of ED might be attributed to hypogonadism [5,6]. The biological mechanism of methadone lies in its ability to block gonadotropin release from the hypothalamus, resulting in significantly reduced in testosterone levels [7]. The effect of methadone on the hypothalamus is mediated by the dopamine pathway [7]. Reduced libido [8,9] and potency [8] as well as low testosterone levels [8,9] are the possible etiological causes of ED in MMT patients. 

The prevalence of ED in the MMT population is high, ranging from 53% to 93% [10,11,12,13,14]. The prevalence of ED increases with age. The prevalence of ED in MMT patients aged >50 years ranges from between 78.8% to 92.9%, higher than that in patients aged <50 years, in whom ED prevalence is 60.0% to 69.3% [11,12]. The high prevalence of ED in the MMT population warrants significant attention for managing the problem.

The effect of ED on quality of life makes it particularly an important matter. In the general population, ED exerts a profound effect on quality of life, as demonstrated in some studies [15,16]. Lugoboni et al. [17] found a significantly better quality of life in opioid substitution therapy (OST) patients without ED. Another study conducted on MMT patients demonstrated that the social domain of quality of life was significantly reduced in MMT patients with ED [11].

Despite the significant impact of ED on quality of life, the treatment-seeking rate for ED remains low. Mercer et al. [18] found that only 10.5% of men with ED had sought help. The low treatment-seeking rate can be explained by several barriers faced by patients. These barriers might vary depending on the population studied. The main barrier that impairs treatment-seeking behavior in the general population in the Western countries is the perception that ED is a part of normal ageing [19]. In contrast, the main barrier that delays treatment seeking for ED in Turkey is embarrassment [20]. The barriers to health-seeking treatment in MMT patients might vary. Despite good knowledge on when and how to access healthcare facilities, the majority of OST patients tend to refrain from seeking medical treatment for physical symptoms [21]. The majority of such patients (64%) had received poor treatment due to drug abuse history or OST [21]. Given the differing challenges in different populations and cultures, it is crucial to determine the health-seeking behavior for ED among MMT patients to understand the characteristics of this specific patient group.

The objective of this study was to determine the health-seeking behavior of MMT patients with ED who perceived themselves as having ED. We aimed to assess the attitudes and health-seeking behavior, the factors associated with treatment-seeking behavior and the effectiveness of the treatment the patients sought based on the patients’ perception. 

## 2. Materials and Methods

### 2.1. Study Design

This was an observational questionnaire-based study. Patients were recruited from six primary health care clinics in Kuala Lumpur, Malaysia, between January to April 2019. The inclusion criteria were male, at least 18 years old, sexually active and had ED for at least 2 months after the initiation of methadone treatment. Patients with a history of ED prior to methadone treatment were excluded, as the association of ED was not related to MMT. Patients who claimed to have ED before the 2 months of MMT were also excluded, as they did not fulfil the criteria of maintenance treatment. Patients who attended primary health care clinics during the study period were invited by a researcher to participate in the study. Patients were recruited using convenience sampling. No payment was given to the patients who participated in this study. 

### 2.2. Ethical Consideration

The study was approved by the Malaysian Ministry of Health Medical Research Ethics Committee (NMRR-18-2586-42421) and the Universiti Kebangsaan Malaysia Research Ethics Committee (UKM PPI/111/8/JEP-2018-622). All patients had provided written informed consent. Patients who had ED were offered referral to a urologist for further assessment.

### 2.3. Instruments

Once informed consent was acquired, patient demographic data on their age, ethnicity, marital status, education level and employment status were obtained. The duration and dose of methadone treatment were also recorded. The patients’ medical records were also referred to for verifying the duration and dose of methadone.

#### 2.3.1. International Index of Erectile Function-5

ED was evaluated using the International Index of Erectile Function-5 (IIEF-5) questionnaire [22]. This instrument had been validated by Rosen, Cappelleri, Smith, Lipsky and Pena [22]. The questionnaire consists of five questions with a 5-point Likert scale. The sensitivity and specificity for discriminating ED at a score of 21 points are 0.98 and 0.88, respectively. The weighted kappa for the IIEF-5 is 0.82. The evaluation was conducted via an interview session with a researcher.

#### 2.3.2. The Health-Seeking Behavior

Health-seeking behavior was evaluated using a validated questionnaire. The questionnaire was validated in a pilot study conducted at the Universiti Kebangsaan Malaysia Medical Centre. The instrument has a percentage agreement of 80% to 100% and kappa coefficient between 0.67 and 1. Patients with ED (based on the IIEF-5 score) were asked about their perception of having ED (self-reported). Patients who thought they had ED were further asked about the effect of ED on their sex life, sources of information on ED and treatment options. The treatment options included medical, self and alternative treatment. Medical treatment included any form of treatment offered by a doctor, such as pharmacological intervention and sex education. Self-treatment included action by the patient, such as taking medicine without prior consultation, herbs, physical exercise, illicit drugs use such as stimulants, or changing methadone dose on their own. Alternative treatments included seeking other practitioners such as traditional masseuses and Chinese medicine practitioners. Patients who sought treatment were also asked about the effectiveness of the treatment as based on their perception. Patients who did not seek medical treatment were queried about their reason for not seeking medical treatment. The health-seeking behavior was evaluated by the researcher in one-to-one interview sessions.

### 2.4. Statistical Analysis

Categorical data are presented as numbers and percentages. Continuous data are presented as the mean and standard deviation (SD) for normally distributed data. If the distribution was not normal, continuous data are presented as the median and interquartile range (IQR). The difference between treatment and non-treatment groups was analyzed using the chi-square test or Fisher’s exact test (if the minimum expected count was <5) for categorical variables. For continuous variables, the independent *t*-test was used to analyze normally distributed variables; the Mann–Whitney test was used for non-normally distributed variables. The statistical analysis was conducted using Statistical Package for the Social Sciences (SPSS), version 22.0 (IBM Corp, Released 2013 IBM SPSS Statistics for Windows Armonk, NY, USA). Missing data in descriptive analysis (health-seeking behavior) were reported as unknown and excluded from inferential analysis (the difference between treatment vs. non-treatment groups). 

## 3. Results

We recruited 335 patients of 433 from the six primary health care clinics. Eighteen patients declined to participate, and 17 patients were not included due to the duration of methadone treatment being less than two months. Out of 300 patients, 146 patients were not sexually active. ED was screened using the IIEF-5. Seventy patients with ED as identified by the IIEF-5 were interviewed on their health-seeking behavior. Two patients were subsequently removed as they did not answer the health-seeking behavior questionnaire. A further 18 patients were further excluded because they perceived themselves as not having ED. Only 50 patients who perceived themselves as having ED were included in the analysis.

### 3.1. Attitudes and Health-Seeking Behaviour

More than half of the patients who thought they had ED reported that their sex life was affected (78%). Most of the patients (48%) did not seek for any information on ED. Only half of the patients (54%) who thought they had ED had attempted treatment, with 66.7% reporting that they opted for self-treatment. Eighty percent of patients who received treatment claimed the treatment was effective. The perception that ‘ED is not a serious condition’ (41.9%) was the main barrier that hindered patients who thought they had ED from seeking treatment (Table 1).

### 3.2. Factors Associated with Treatment-Seeking Behaviour

In terms of demographic and methadone treatment (dose and duration) variables, education level (*p* = 0.017) and marital status (*p* = 0.008) were the only significant predictive factors associated with health-seeking behavior between patients with ED who sought and did not seek treatment (Table 2). 

### 3.3. Effectiveness of ED Treatment

Table 3 shows the types of treatment and the effectiveness of treatment sought by the MMT patients. There were no significant differences between the effectiveness of medical, self and alternative treatment based on patients’ perception (*p* = 0.770).

## 4. Discussion

The proportion of patients who were not sexually active was high (48.7%). We did not explore the reason behind the sexual inactivity. Reduced libido and low testosterone level are the possible reasons. In the male aging population, reduced libido is strongly associated with low testosterone levels [23]. This result is further supported by the finding that men with low testosterone levels on testosterone replacement therapy had significantly increased libido [24]. Moreover, Bliesener et al. [8] showed that MMT patients had profoundly lower testosterone levels and higher levels of sexual dysfunction (in terms of libido and potency) as compared to a buprenorphine maintenance treatment (BMT) group. In addition, the level of testosterone hormone in the BMT group did not differ significantly as compared to that of the control group [8]. Another possible cause of sexual inactivity is the avoidance of sexual activity secondary to the sexual problem [17].

Most of the patients (48%) did not refer to any source for ED information. This result differs from that of Zhang et al. [25]. Zhang, Yu, He and Jin [25] showed that, among primary health care patients with ED in China, physicians (54%) were the most frequently consulted source, followed by the internet (52%) and friends (34%). Only 6% of ED patients did not refer to any source [25]. Among ED patients in primary healthcare who had talked to someone about their ED condition, partners (54.1%) were the most frequent source of ED information, followed by doctors (32.4%) and friends (6.3%) [26]. However, in that study [26], other sources of reference (internet, media) were not explored. These data show that MMT patients tend to keep the problem to themselves without referring to any source.

Here, more than half of the patients (54%) had sought ED treatment of any kind. However, only 8% of the patients had ever discussed their problem with a doctor. The perception of ED as not serious (41.9%) was the main barrier to MMT patients not seeing doctors, followed by hesitancy (32.6%). A primary healthcare study reported that only 10.5% of men with ED had consulted a doctor on their ED problem [26]. Ab Rahman, Al-Sadat and Yun Low [26] reported that 38.2% of men felt uncomfortable discussing ED with their doctor. In contrast, a study conducted in the US, UK, France, Germany, Italy and Spain demonstrated that a total of 46% of men had sought treatment from a doctor [19]. The main barriers preventing patients from seeking treatment were the belief that ED is a normal part of the ageing process (43.6%), followed by anticipating that ED would resolve on its own (31.4%) and embarrassment (26.9%) [19]. The Men in Australia Telephone Survey (MATeS) study showed that 58% of patients had consulted their doctors regarding ED [27]. However, the study did not explore the barriers associated with health-seeking behavior. The findings show variations in patient preference in different populations. 

Education level was a significant factor that determined the treatment-seeking behavior in the present study. Table 2 shows that men with higher education levels had a greater tendency to seek treatment compared to men with lower education levels. The possible reason is that patients with higher education levels tend to have higher levels of awareness and elevated perspective regarding ED. Gülpinar, Haliloğlu, Abdulmajed, Bogğa and Yaman [20] showed that ED patients with lower education levels took a longer time to seek treatment. 

Marital status was another significant risk factor associated with treatment-seeking behavior. Our results show that patients who sought treatment were either married or divorced. Papaharitou et al. [28] found that being in a stable relationship was significantly associated with previous consultation with a doctor. In contrast, Shabsigh, Perelman, Laumann and Lockhart [19] found that being widowed was a barrier to seeking treatment. The difference might be due to the difference in methodology. Here, we classified ED based on the IIEF-5, which requires men to have a stable partner for at least six months. Shabsigh, Perelman, Laumann and Lockhart [19] classified ED based on one question regarding difficulty in attaining or maintaining erection. The assessment of ED by Shabsigh, Perelman, Laumann and Lockhart [19] did not specify the need for a partner or sexual intercourse. The difference in assessment might explain the conflicting findings between the studies.

In the present study, the most favored treatment was self-treatment. However, the effectiveness of the treatment obtained was not significantly different and this might be attributed to the small sample size. The specific measures taken in each category were not explored further, as the sample size is small and there were various treatment options in each main treatment category. Ab Rahman, Al-Sadat and Yun Low [26] reported that, among patients who attended primary health care clinics for various reasons, the primary treatments were the herbal medicine *tongkat ali* (40%), followed by phosphodiesterase-5 inhibitor (35%). However, the study did not explore the perception of patients toward the effectiveness of the treatment taken. 

In summary, ED affects the sex life of the majority of MMT patients. MMT patients tend to keep their ED problem to themselves. More than half of the patients had sought treatment, with the majority opting for self-treatment and the difference in the effectiveness among the treatment types was not significant. The main barriers to seeking treatment were the perception of ED as not a serious problem and hesitancy. The determinants for seeking treatment were higher education level and being married or divorced. ED may induce stressful conditions that may affect health-seeking behavior. Stress can induce both physical and psychological changes that determine health-seeking behavior [29]. Psychological changes such as anxiety often spur a person to take action to reduce the stress level. [29] Depending on the person previous experienced, the outcome of health behavior may be beneficial such as after consulting a doctor, or detrimental, such as abusing stimulants. In the OST population, negative experiences such as poor treatment [21] and discrimination [30] may prevent patients from seeking appropriate treatment. A holistic approach must be formulated to overcome the issues related to health-seeking behavior in the MMT population.

Several interventions are recommended. Doctors and staffs involved in the methadone clinic service should actively screen ED at initiation of MMT and periodically during follow-up. Moreover, the policymakers should make ED screening as a part of integrated assessment in the MMT protocol, as the problem is prevalent in opioids users. Methadone facilities should organize talks or small-group discussions on the problems faced by MMT patients. ED should be included as part of the discussion topic. Such talks or discussions would allow patients to share their concerns and barriers regarding certain problem. The feedback received from the patients should be discussed further to overcome the issues and improve service delivery. Moreover, practitioners can take the opportunity to talk about ED, the available treatment options as well as the adverse effect of harmful self-treatment or alternative treatment. Doctors should maximize their encounters with patients during routine follow-up to establish good rapport and trust with their patients. We hope that these measures will increase patient knowledge and change their perceptions and their health-seeking behaviors.

The limitation of this study was the small sample size, as almost half of the patients were sexually inactive. Moreover, we could only assess the effectiveness of the treatment based on patients’ perception. A structured assessment is needed to assess the effectiveness of each treatment appropriately to avoid misleading findings on which treatment is effective. As the study was a cross-sectional design, we could not determine a temporal relationship. The results must be interpreted with caution, as we only conducted univariate analysis. The confounding factors could not be controlled, as we could not perform multivariate analysis due to the small final sample number. The strength of this study was the focus of health-seeking behavior on this specific population (methadone maintenance patients). ED is a sensitive issue, and it is prevalent in this population. Therefore, it is crucial to study the behavior that might differ from that of the general population.

## 5. Conclusions

The health-seeking rate for medical treatment among MMT patients with ED needs to be improved. Measures for increasing awareness of ED among MMT patients should be taken to overcome the barriers to health-seeking behavior. Health practitioners should take action to screen ED in this population to increase the detection rate and to offer appropriate management according to the patients’ needs.

## Figures and Tables

**Table 1:** The health-seeking behavior for erectile dysfunction (ED) among methadone maintenance treatment (MMT) patients (*n* = 50).

Question	Number (Percentage)
Do you think that ED affects your sex life?	
Yes	39 (78.0)
No	11 (22.0)
Which sources did you refer to for information on your problem?	
Friends	12 (24.0)
Partner	7 (14.0)
Doctor	4 (8.0)
Pharmacist	1 (2.0)
Social media	1 (2.0)
Internet	1 (2.0)
None	24 (48.0)
Treatment status	
Yes	27 (54.0)
No	23 (46.0)
Which treatment did you choose for your problem? (Respondents who sought treatment, *n* = 27)	
Medical	5 (18.5)
Self	18 (66.7)
Alternative	4 (14.8)
Do you think that the treatment improved your condition?	
Yes	20 (80.0)
No	5 (20.0)
Unknown	2
Why did you not consult the doctor regarding your condition? (Respondents who did not seek medical treatment, *n* = 45)	
ED is not a serious condition	18 (41.9)
Hesitant to talk about ED	14 (32.6)
The doctor is not interested	3 (7.0)
Worried about adverse effects of medication	3 (7.0)
Satisfied with self-treatment	3 (7.0)
Could buy the medication at a pharmacy	1 (2.3)
No channel	1 (2.3)
Unknown	2

ED: Erectile dysfunction.

**Table 2:** Bivariate analysis of the associations between demographic and methadone treatment (dose and duration) factors and health-seeking behavior (treatment status) among MMT patients with ED (*n* = 50).

Characteristic	Treatment Status		*p*-Value
	Yes	No	
N (%)	27 (54.0)	23 (46.0)	
Age (years), mean (SD)	47.3 (7.5)	51.0 (10.2)	0.151 ^†^
Methadone dose (mg), mean (SD)	55.4 (26.8)	62.4 (32.9)	0.410 ^†^
MMT duration (months) ^a^, median (IQR)	43 (21-64)	31 (14-60)	0.235 ^‡^
Ethnicity, *n* (%)			
Malay	24 (54.5)	20 (45.5)	1.000 ^#^
Non-Malay	3 (50.0)	3 (50.0)	
Marital status, *n* (%)			
Single	0 (0.0)	5 (100.0)	0.008 ^#,**^
Married	25 (58.1)	18 (41.9)	
Divorced	2 (100.0)	0 (0.0)	
Employment status, *n* (%)			
Employed	24 (60.0)	16 (40.0)	0.155 ^#^
Unemployed	3 (30.0)	7 (70.0)	
Level of education, *n* (%)			
Primary	0 (0.0)	5 (100.0)	0.017 ^#,*^
Secondary	24 (58.5)	17 (41.5)	
Tertiary	3 (75.0)	1 (25.0)	

^a^ Indicates that the distribution is skewed. ^†^ Independent *t*-test. ^‡^ Mann–Whitney test. ^#^ Fisher’s exact test. * *p* < 0.05 and ** *p* < 0.01. ED: Erectile dysfunction; IQR: interquartile range; MMT: methadone maintenance treatment; SD: standard deviation.

**Table 3:** The type of treatment taken and the effectiveness of the treatment according to patients’ perception (*n* = 25).

Choice of Treatment, *n* (%)	Perception of Treatment Effectiveness		*p*-Value
	Yes	No	
Medical	2 (66.7)	1 (33.3)	0.770 ^#^
Self	15 (83.3)	3 (16.7)	
Alternative	3 (75.0)	1 (25.0)	

^#^ Fisher’s exact test.
